# *Zanthoxylum zanthoxyloides* Alkaloidal Extract Improves CCl_4_-Induced Hepatocellular Carcinoma-Like Phenotypes in Rats

**DOI:** 10.1155/2021/3804379

**Published:** 2021-07-21

**Authors:** Desmond Omane Acheampong, Isaac Kyei Baffour, Victor Yao Atsu Barku, Justice Kwaku Addo, Mainprice Akuoko Essuman, Alex Boye

**Affiliations:** ^1^Department of Biomedical Sciences, School of Allied Health Sciences, College of Health and Allied Sciences, University of Cape Coast, Cape Coast, Ghana; ^2^Department of Chemistry, School of Physical Sciences, College of Agriculture and Natural Sciences, University of Cape Coast, Cape Coast, Ghana; ^3^Department of Medical Laboratory Science, School of Allied Health Sciences, College of Health and Allied Sciences, University of Cape Coast, Cape Coast, Ghana

## Abstract

**Background:**

Despite the enrollment of new small molecules such as Sorafenib for the treatment of hepatocellular carcinoma (HCC), HCC still remains a significant contributor to cancer-related mortality and morbidity globally. *Zanthoxylum zanthoxyloides* is long suspected of possessing anticancer bioactive compounds that may hold the prospect of adjunctive therapy against inflammation-related cancers such as HCC.

**Objective:**

This study assessed the effects of an alkaloidal extract of the leaves of *Zanthoxylum zanthoxyloides* on CCl_4_/olive oil (1 : 1 v/v)-induced HCC-like phenotypes in rats.

**Materials and Methods:**

*Zanthoxylum zanthoxyloides* alkaloidal extract (ZZAE) was prepared using Soxhlet and liquid-liquid extraction methods. Subsequently, ZZAE was characterized phytochemically. In the curative method, experimental HCC was established in adult (8–10 weeks old) male Sprague-Dawley rats weighing 150–300 g by twice-daily administration of CCl_4_/olive oil (1 : 1 v/v) (2 mL/kg *ip*). After confirmation of experimental HCC in rats, the rats were randomly reassigned into seven (7) groups of seven (7) rats each and treated daily for 12 weeks as follows: control (normal saline, 5 ml/kg *po*), model (CCl_4_, 5 ml/kg, *ip*), ZZAE (50, 100, and 200 mg/kg *po*), carvedilol (6.25 mg/kg *po*), and 20% Tween20 (1 mL/rat, *po*). To assess whether ZZAE has a prophylactic (preventive) effect, rats were first treated with ZZAE and later exposed to CCl_4_ reconstituted in olive oil.

**Results:**

ZZAE (100 and 200 mg/kg) and carvedilol decreased tumor incidence compared to that of control. Compared to control, ZZAE (100 and 200 mg/kg) significantly (*P* < 0.05) improved serum GGT. Compared to control, ZZAE improved hepatohistological distortions induced by CCl_4_/olive oil and also improved liver/body weight ratio. Compared to water, ZZAE arrested mitosis in the *Allium cepa* assay.

**Conclusion:**

ZZAE ameliorated CCl_4_/olive oil-induced HCC-like phenotype in rats and demonstrated general hepatoprotective effects by improving liver and kidney function markers. This finding rationalizes the need for further studies on ZZAE as a potential source of bioactive anti-HCC compounds.

## 1. Introduction

Cancer is one of the debilitating systemic diseases that afflict millions of people worldwide [1, 2]. Hepatocellular carcinoma (HCC) is the third leading cause of death in all cancer-related mortalities globally, with about 750,000 incidences and 700,000 deaths reported annually [3]. Hepatitis, drug/chemical poisoning, steatosis, heavy alcohol consumption, pheochromocytoma, and aflatoxin-related food poisoning remain major risk factors for HCC [4]. Advancements in medicine have inured some gains in cancer treatment and management and specifically improved screening and prognosis for some specific cancer subtypes. Cancer treatment and management remain poor as evidenced by poor survival rates, high recurrence rate, and serious treatment-related complications [5]. Many anticancer therapies including chemotherapy, pharmacotherapy, radiotherapy, immunotherapy, surgical resection, and chemoembolization (e.g., transarterial chemoembolization), just to mention but a few, have all been used to manage various cancers including HCC without significant success in terms of survival rates and quality of life after treatment [2, 6]. Sorafenib, a multikinase inhibitor was approved by the FDA for the treatment of cancer including HCC, but just like the other therapies, it is also identified with many adverse events [7]. These problems associated with cancer treatment and management have been attributed to many factors including multidrug resistance of tumor cells, damage to normal cells due to nonspecific anticancer therapies (e.g., chemotherapy and radiotherapy), multifactorial and complex pathogenesis of cancer, and ineffective conventional therapies [8]. It is in response to this that attention has now shifted to the exploration of novel bioactive compounds from natural products, specifically medicinal plants, which have been the mainstay of the pharmaceutical subindustry as templates for pharmaceutical semisynthesis of therapeutic agents. This shift has been occasioned by recent reports, including that of the World Health Organization (WHO) attesting to the popular use of herbal medicine as an alternative therapy for the management of various diseases including cancer in both developed and developing countries of the world. Indeed, the dominance of natural products, specifically herbal medicine in the treatment/management of human diseases, is unmatched. It was reported that plant species in the genus *Zanthoxylum* are rich in bioactive anticancer compounds [9, 10]. Previously, the anti-inflammatory and gastroprotective effects of *Zanthoxylum zanthoxyloides* were demonstrated [9, 11, 12]. On the basis of these reports, many studies have investigated some bioactivities of *Zanthoxylum zanthoxyloides* including antiplasmodial [13], antitrypanosomal [14], antimalarial [15], antihelminthic [16], antisickling [17], and diversity of the genus [18]. Also, it was shown that *Zanthoxylum zanthoxyloides* contain diverse phytocompounds including flavonoids, tannins, coumarins, fluoroquinoline, oxylipin, aromatic compounds, acridone alkaloids, fluoride, and zantholic acid [19–25]. The current study proposed and tested the hypothesis “Herb-derived anti-inflammatory agents have an inherent anticancer effect on inflammation-related cancers such as HCC,” by investigating anti-HCC and hepatoprotective effects of *Zanthoxylum zanthoxyloides* alkaloidal extract (ZZAE) using experimental HCC in rats. Interestingly, our preliminary results showed that ZZAE has efficacy against experimental HCC by decreasing tumor burden (tumor volume and tumor multiplicity), increasing the survival rate of rats, and improving liver function markers.

## 2. Materials and Methods

### 2.1. Drugs and Chemicals

Carvedilol (Bazayan & Co., Zurich, Switzerland), CCl_4_ (El Gomhorya Co., Cairo, Egypt), olive oil (AddPharma Ghana Limited, Accra, Ghana), and Tween20, ethanol, and petroleum spirit (Thermo Fisher Scientific, Massachusetts, USA) were used. All other chemicals and reagents used in this study were of analytical grade. All animal procedures and extraction processes were carried out at the laboratories of the Department of Biomedical Sciences, UCC. Tissue processing, hematological analysis, and biochemical assays were carried out at Euracare Advanced Diagnostic Center (Accra, Ghana).

### 2.2. Collection, Identification, and Authentication of the Plant Material

The aerial parts of *Zanthoxylum zanthoxyloides* were collected from Adisadel, a suburb of Cape Coast, Central Region of Ghana, on August 17, 2018. The plant parts were identified and authenticated by Mr. Francis Otoo, the Curator at the herbarium unit, School of Biological Sciences, University of Cape Coast, where a voucher specimen (SC/SBS/UCC/43BSH) was deposited.

### 2.3. Preparation of *Zanthoxylum zanthoxyloides* Crude Extract (ZZE)

The leaves of *Zanthoxylum zanthoxyloides* were washed with tap water and air-dried for 3 weeks. The dried leaves were pulverized using a hammer mill (Polymix Micro Hammer Cutter Mill, Glen Mills Inc., USA). A 4.37 kg quantity of the resultant powdered leaves was defatted with 7 L of petroleum ether at 60–80°C. A crude extraction was performed with 6 L of 70% ethanol in a Soxhlet apparatus (L3 Soxhlet extractor, Ergotech Soxhlet Apparatus Co., UK). A 1,542 mL quantity of the extract was evaporated on a water bath (Premiere HH-4 Digital Water Bath, C & A Scientific Co. Inc., USA). Subsequently, the resultant extract was dried in a crucible in a 40°C hot air oven (Oven 300 plus series, Gallenkamp, England) over 24 h yielding 762.84 g of ZZE. The ZZE was stored in a refrigerator at 4°C until use.

### 2.4. Preparation of *Zanthoxylum zanthoxyloides* Alkaloidal Extract (ZZAE)

The alkaloidal extraction procedure was carried out as previously described [26]. Briefly, ZZE was acidified with 15% acetic acid and thoroughly shaken to ensure the conversion of all free forms of alkaloids into salt form. Chloroform was added to the acidified ZZE and allowed to stay for 6 hours. The aqueous layer from the previous step was basified with 10% ammonia and thoroughly shaken and allowed to stay overnight. The organic layer was extracted with chloroform until there was no color formation, indicating extraction of alkaloidal extract fraction (149.45 g) and named and used in this study as ZZAE. To confirm alkaloids, a confirmatory test was conducted.

### 2.5. A High-Performance Liquid Chromatography (HPLC) Coupled with Diode Array Detector Analysis on ZZAE

The analysis was carried out using Varian Prostar 210 Galaxie system with diode array detector. The analytical conditions included solvent systems used for elution, which were mobile phase A (methanol with 0.01% formic acid), mobile phase B (10% ammonium acetate with 0.01% formic acid), and elution solvent mixture A : B (10 : 90% v/v). The LC system was operated in isocratic mode with a flow rate of 1.5 mL/min. The column used was id: Bondclone, 10 u, C-18 300X 3.90 made by Phenomenex, and it was maintained at a temperature of 40°C in an oven. The diode array detector was operated in dual-wavelength mode, that is, 254 nm (channel 1) and 280 nm (channel 2). The flow cell temperature was maintained at 40°C. The injection volume for the sample was 0.5 *μ*L and the total run and detection time was 40 minutes.

### 2.6. Animal Acquisition and Husbandry

Eighty (80) healthy adult (8–10 weeks old) male Sprague-Dawley rats (150–300 g) were purchased from Noguchi Memorial Institute of Medical Research (NMIMR), University of Ghana. The rats were kept at the animal house of the School of Biological Sciences under ambient conditions of temperature, pressure, and light/dark cycle. Rats were housed in aluminum cages (12.5 × 16.6 × 7.5 inches) with softwood shavings as bedding. Rats were allowed two weeks to acclimatize with laboratory conditions before all experiments began. Rats had access to pelleted rodent chow (Gafco, Tema, Ghana) and water *ad libitum*. These conditions were varied to meet the specific requirements of some experiments. All animal experiments, procedures, and techniques used in this study were in full compliance with institutional, national, and international (the National Institute of Health Guidelines for Care and Use of Laboratory Animals, NIH publication No. 85–23, revised 1985) guidelines on the use of animals in scientific experimentation.

### 2.7. Establishment of CCl_4_/Olive Oil-Induced HCC in Rats

CCl_4_-induced HCC in rats was established according to a previously described method [27]. Briefly, out of the 80 rats, 62 were administered with CCl_4_/olive oil (1 : 1 v/v) (2 ml/kg *ip*) twice a week for 16 weeks. Three rats were randomly selected, sacrificed under chloroform anesthesia, liver isolated, and histologically assessed to confirm or otherwise establish experimental HCC.

### 2.8. Grouping and Dosing

Except rats in control, prophylactic ZZAE, and ZZAE only groups, all confirmed CCl_4_-induced HCC rats were reassigned into various groups and treated as follows.

#### 2.8.1. Control Group

Rats were treated with normal saline (5 ml/kg *po*) + access to rodent chow and water *ad libitum*.

#### 2.8.2. Model Group

Rats received CCl_4_/olive oil (1 : 1 v/v) (2 ml/kg *ip*) in the morning twice a week for 16 weeks + no treatment + access to rodent chow and water *ad libitum*.

#### 2.8.3. Carvedilol Group

Rats received CCl_4_/olive oil (1 : 1 v/v) (2 ml/kg *ip*) in the morning twice a week for 16 weeks + carvedilol (6.25 mg/kg *po*) in the afternoon + access to rodent chow and *water ad libitum*.

#### 2.8.4. Tween20 Group

Rats received CCl_4_/olive oil (1 : 1 v/v) (2 ml/kg *ip*) in the morning twice a week for 16 weeks + 20% Tween20 (1 mL/rat) once daily in the afternoon + access to rodent chow and *water ad libitum*.

#### 2.8.5. ZZAE (50 mg/kg) Group

Rats received CCl_4_/olive oil (1 : 1 v/v) (2 ml/kg *ip*) in the morning twice a week for 16 weeks + ZZAE (50 mg/kg *po*) + access to rodent chow and *water ad libitum*.

#### 2.8.6. ZZAE (100 mg/kg) Group

Rats received CCl_4_/olive oil (1 : 1 v/v) (2 ml/kg *ip*) in the morning twice a week for 16 weeks + ZZAE (100 mg/kg *po*) + access to rodent chow and *water ad libitum*.

#### 2.8.7. ZZAE (200 mg/kg) Group

Rats received CCl_4_/olive oil (1 : 1 v/v) (2 ml/kg *ip*) in the morning twice a week for 16 weeks + ZZAE (200 mg/kg *po*) + access to rodent chow and *water ad libitum*. Treatments were done daily for a period of 16 weeks.

#### 2.8.8. Prophylactic ZZAE Group

Rats received ZZAE (200 mg/kg *po*) daily in the morning + CCl_4_/olive oil (1 : 1 v/v) (2 ml/kg *ip*) twice a week for 16 weeks in the afternoon + access to rodent chow and *water ad libitum*.

#### 2.8.9. ZZAE Only Group

Rats received ZZAE (200 mg/kg *po*) daily for 16 weeks + access to rodent chow and *water ad libitum*.

### 2.9. Body Weight Measurements

Body weight of rats was measured weekly and doses were adjusted to reflect body weight changes. Prior to sacrifice, rats were weighed and anesthetized. Mean change in body weight was determined for all groups.

### 2.10. Blood Collection and Isolation of Livers

Rats were sacrificed under chloroform anesthesia at the end of the 16th week and blood was collected into labeled EDTA tubes for hematological (full blood count) and biochemical (liver and kidney enzymes assays) assessments. The liver of each rat was isolated surgically. Wet weight of freshly isolated livers was measured and representative liver lobes were preserved in 10% formalin for histological assessment. The mean liver/body weight ratio was determined for each treatment group.

### 2.11. Measurement of Hematological and Biochemical Parameters

Full blood count (FBC) was profiled for each group after blood collection using an automated hematology analyzer (MAXM Analyzer C23644-DxH, California, USA). To monitor liver enzymes and kidney biomarkers, blood was centrifuged at 15000 rpm for 8 min, followed by serum separation. Liver enzymes and kidney biomarkers were measured using an automated biochemical analyzer (Hitachi Model 917, Roche Diagnostics, Indianapolis, IN).

### 2.12. Estimation of Tumor Multiplicity

Tumor multiplicity was measured according to a previous method [28]. Briefly, representative livers for each group were macroscopically and microscopically examined by three independent investigators and the number and size of tumor nodules were determined. The mean for each group was estimated and used to determine tumor multiplicity for each group.

### 2.13. Histological Assessment of Liver Tissues

Liver tissue sections were prepared per a previously described method [29] with some modifications. Tissue samples from the liver were fixed in 10% neutral buffered formalin, dehydrated in increased concentrations of alcohol, cleared in xylene, and embedded in paraffin. Semiserial 4 micrometers (*μ*m) sections (microtome HM-355S Automatic Microtomes Thermo Scientific) were stained with Harris hematoxylin and eosin (H&E) and permanently mounted on microscopic slides using DPX and coverslips and then observed under a light microscope for the investigations of any histological change. Images were captured on an optical microscope (Zeiss, Germany) coupled to a high-resolution camera (AmScope, California) and analyzed using the AmScope Software 2020.

### 2.14. *Allium cepa* Root Tip Assay

Cytotoxicity of ZZAE was assessed using *Allium cepa* root tip cells as previously described [30, 31], with some modifications. Briefly, *Allium cepa* bulbs of approximately the same sizes were obtained, the outer scales were removed, and the dead root tips were carefully scraped off. The bulbs were then grown in a 24-hour dark cycle for 48 hours with the root meristems suspended in tap water at ambient temperature until the roots had grown to approximately 1 cm. Excess water on the onion root tips was gently cleaned with tissue paper and incubated for 72 h in each of the following: control (tap water only), carvedilol (1 mg/mL), and ZZAE (0.1 and 1 mg/mL). After 72 hours, the number and the length of roots from onion bulbs in each group were measured. Slides were made from onion root tip cells for examination under a microscope.

### 2.15. Statistical Analysis

Data were analyzed using GraphPad Prism 7 software (GraphPad Software, San Diego, Califonia, USA). Data were expressed as mean ± standard deviation (SD). Mean comparison between groups was done using a one-way analysis of variance (ANOVA). Differences between means were analyzed using Dunnett's multiple comparison tests. *P* ≤ 0.05 was considered statistically significant in all analyses.

## 3. Results

### 3.1. ZZAE Yield, Phytochemical Composition, and HPLC Chromatographic Analysis

**Figure 1:**
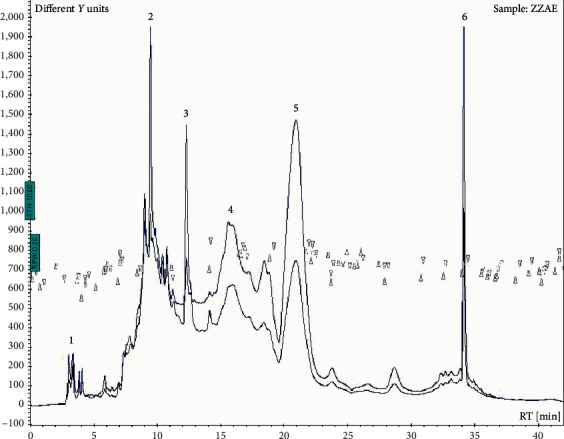
HPLC chromatogram of ZZAE. ZZAE: *Zanthoxylum zanthoxyloides* alkaloidal extract.

Table 1 shows the % yield of *Zanthoxylum zanthoxyloides* extract (ZZE) and *Zanthoxylum zanthoxyloides* alkaloidal extract (ZZAE). Preliminary screening of ZZE revealed the presence of carbohydrates, tannins, alkaloids, glycosides, and terpenoids (Table 2). Analysis of ZZAE using HPLC showed 6 peaks (Figure 1).

### 3.2. Effect of ZZAE on Body Weight and Survival of Rats

**Figure 2:**
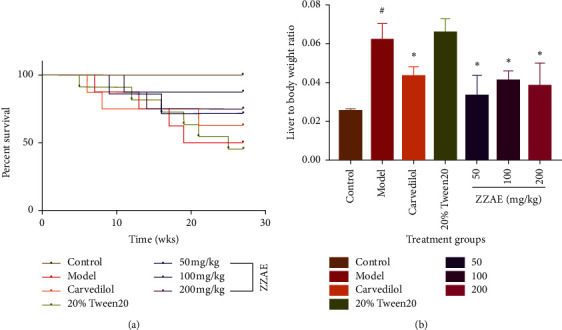
(a) Effect of ZZAE on survival rats exposed to CCl_4_/olive oil over a 16-week period. (b) Effect of ZZAE on liver/body weight ratios. Each value is the mean ± SD, *n* = 4. ^#^*P* ≤ 0.05 (control versus model groups); ^*∗*^*P* ≤ 0.05 (treatments versus model groups); ZZAE: *Zanthoxylum zanthoxyloides* alkaloidal extract.

The control group had on average 50% mean body weight gain compared to 14.7% for the model group. However, treatment of CCl_4_-induced HCC rats with ZZAE (50, 100, and 200 mg/kg) improved the mean % body weight gain compared to the model group, particularly the ZZAE (100 mg/kg) (Table 3**).** The control group recorded 100% survival while the model group recorded a survival rate of 50%. Relative to the model group, the ZZAE group improved survival rate >75% (Figure 2(a)**)**. Compared to the control group, model and Tween20 groups had increased liver/body weight ratios. Relative to the model group, ZZAE and carvedilol groups produced a significant (*P* ≤ 0.05) decrease in liver/body weight ratios (Figure 2(b)).

### 3.3. Effect of ZZAE on Tumor Incidence

**Figure 3:**
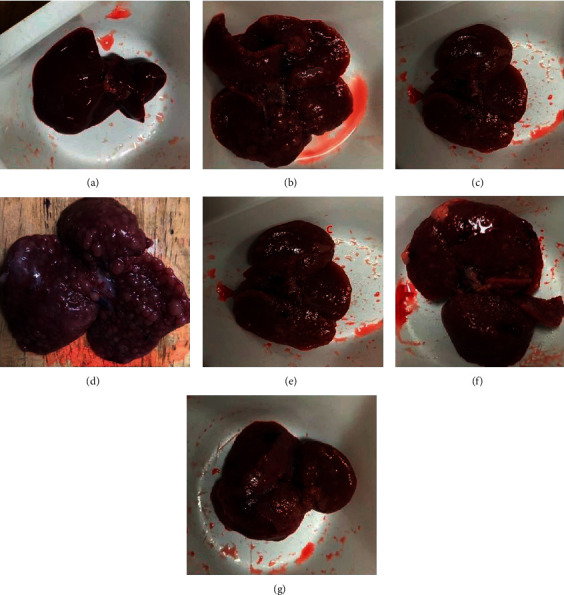
Effect of ZZAE on gross liver anatomy. (a) Control group, (b) model group, (c) carvedilol group (6.25 mg/kg), (d) Tween20 (vehicle) group showed inflamed liver and diffused dysplastic nodules, (e) ZZAE group (50 mg/kg), (f) ZZAE group (100 mg/kg), and (g) ZZAE group (200 mg/kg). ZZAE: *Zanthoxylum zanthoxyloides* alkaloidal extract.

As expected, the control group had no tumors. Relative to control, the model group showed high tumor incidence. Relative to the model group, ZZAE demonstrated efficacy particularly ZZAE groups (100 and 200 mg/kg) (Figure 3 and Table 4**).**

### 3.4. Effects of ZZAE on CCl_4_/Olive Oil-Induced Hepatohistological Distortions

**Figure 4:**
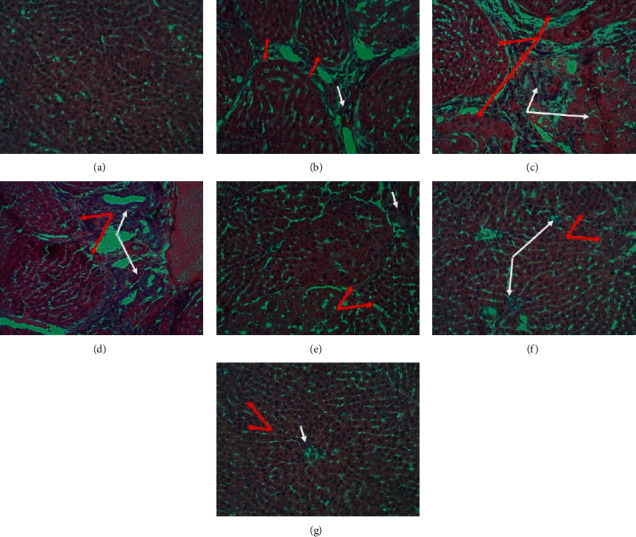
Effect of treatments on liver histology. (a) Control group, (b) model group, (c) carvedilol group (6.25 mg/kg), (d) 20% Tween20 (vehicle) group, (e) ZZAE group (50 mg/kg), (f) ZZAE group (100 mg/kg), and (g) ZZAE group (200 mg/kg). ZZAE: *Zanthoxylum zanthoxyloides* alkaloidal extract.

Figure 4 shows H&E stained histomicrographs of liver sections for the control, model, and treatment groups. Control rats showed normal hepatocytes with polygonal shapes and tightly parked, with basophilic central nuclei. The nuclei were separated by hepatic sinusoids and Kupffer cells were inactivated within the walls of the sinusoids. Unlike the control, the model group demonstrated degenerative changes and swelling of some hepatocytes, vacuolated cytoplasm, and shrunken nuclei. Hepatic cells were surrounded by abnormal reticulin network (red arrows) and large amounts of fatty pigments and a large number of necrotic cells (white arrows). Also, cells were loosely packed with homogeneous hypereosinophilic cytoplasm. Compared to the model group, the ZZAE group demonstrated reversal of hepatic damage and restoration of hepatic architecture. The carvedilol group revealed liver neoplastic cellular alteration (red arrows), enlarged hepatocytes with apparent pleomorphisms and necrotic cells (white arrow), and enlarged hepatic nuclei. Cells are surrounded by an abnormal reticulin network. The 20% Tween20 group revealed hepatocyte pleomorphism and degenerative changes in cells surrounded by abnormal reticulin network and fatty pigments (red arrows). Some cells were loosely packed, necrotic, and undifferentiated (white arrow).

### 3.5. Effect of ZZAE on Liver Markers

**Figure 5:**
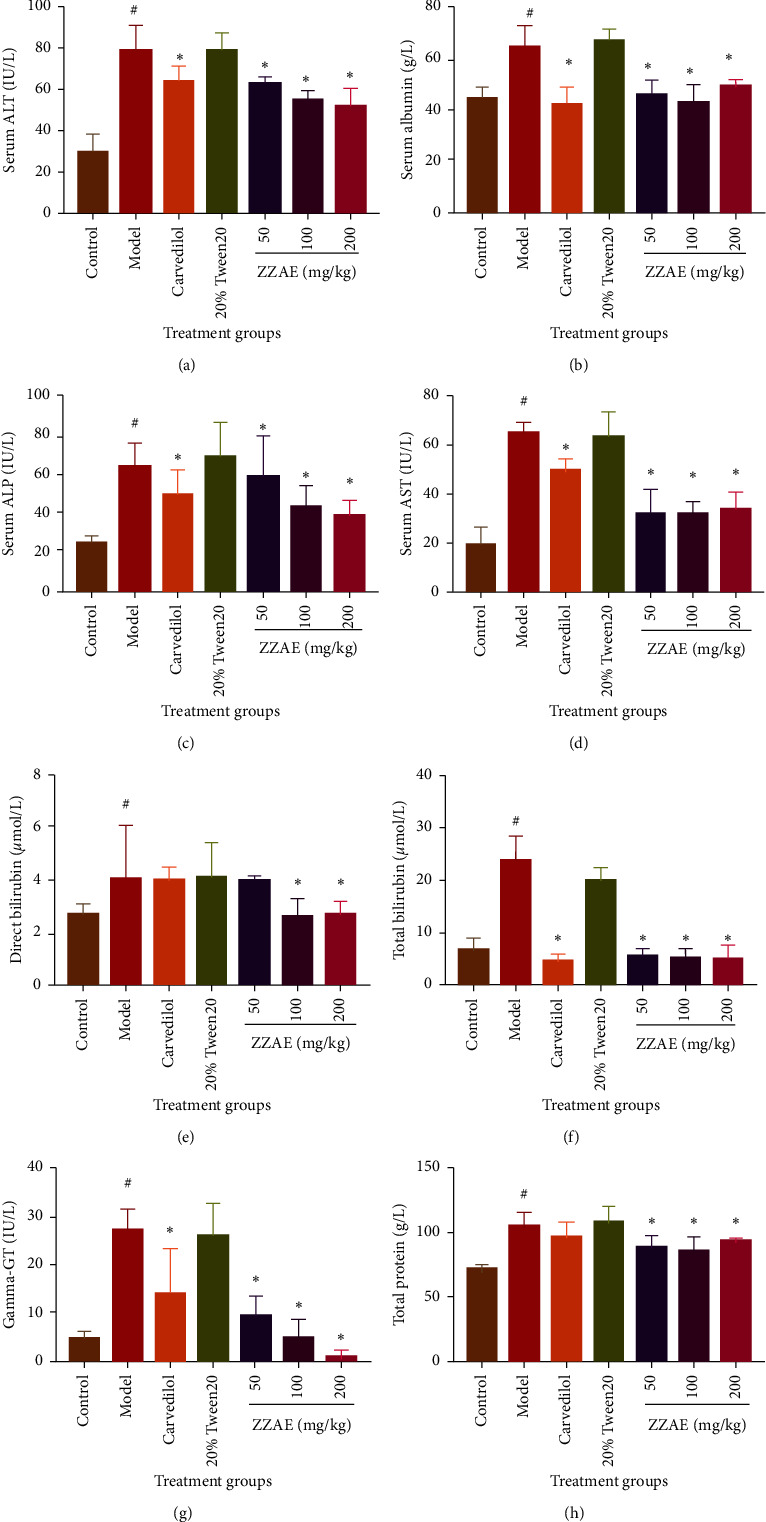
Effect of ZZAE on liver markers. (a) Serum alanine transaminase (ALT), (b) serum albumin, (c) serum alkaline phosphatase (ALP), (d) serum aspartate transaminase (AST), (e) direct bilirubin, (f) total bilirubin, (g) gamma-glutamyltransferase (GGT), and (h) total protein. Each value is the mean ± SD, *n* = 4. ^#^*P* ≤ 0.05 (control versus model group); ^*∗*^*P* ≤ 0.05 (treatments versus= model). ZZAE: *Zanthoxylum zanthoxyloides* alkaloidal extract.

Compared to the control group, the model group demonstrated elevated levels of liver enzymes and markers. ZZAE and carvedilol significantly (*P* < 0.05) reduced the elevated levels of liver enzymes and markers (Figure 5**)** compared to that of the model group. However, the decrease in the levels of ALP of ZZAE (50 mg/kg) group was statistically insignificant. Except total protein, ZZAE produced a dose-dependent decrease in elevated liver enzymes.

### 3.6. Effect of ZZAE on Kidney Markers

**Figure 6:**
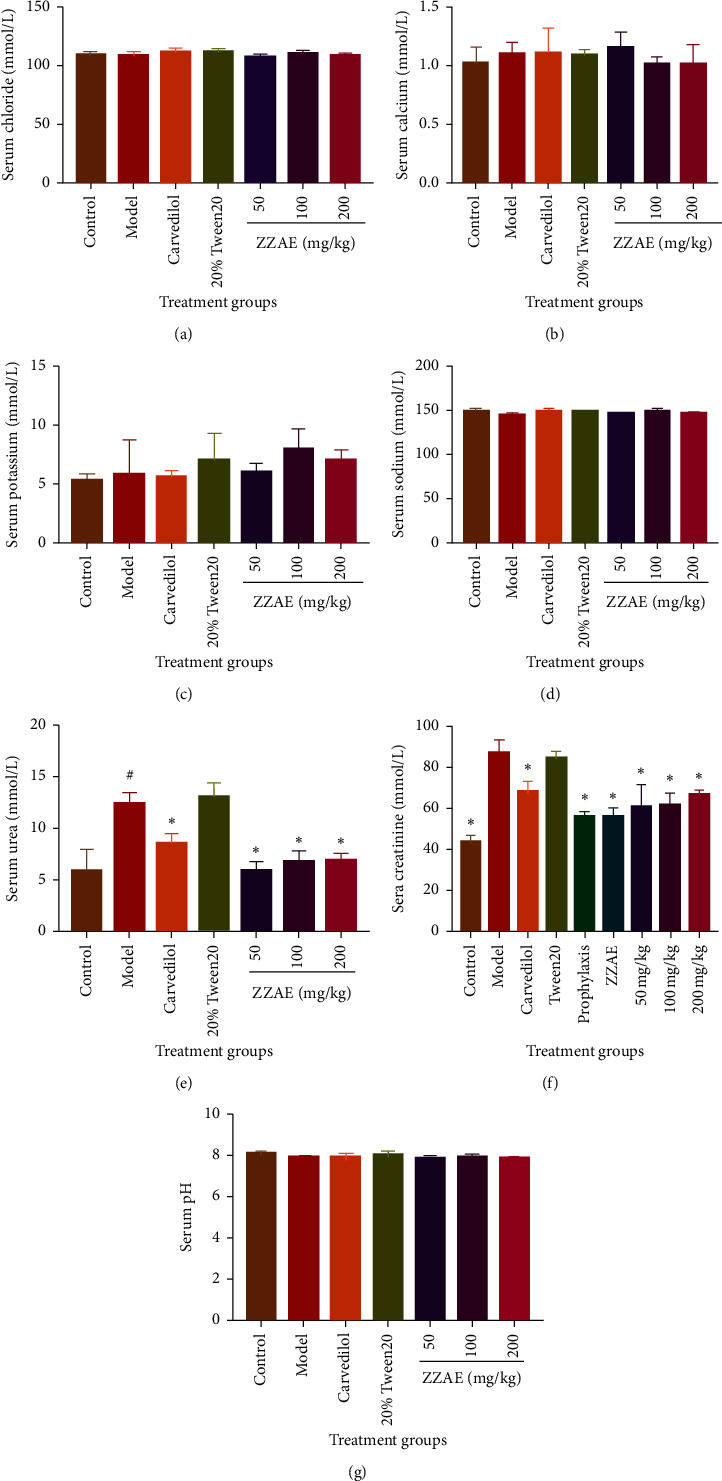
Effect of ZZAE on kidney markers. (a) Chloride (Cl^−^), (b) calcium (Ca^2+^), (c) potassium (K^+^), (d) sodium (Na^+^), and (e) urea. Each value is the mean ± SD, *n* = 4; ^#^*P* ≤ 0.05 (control versus model group); ^*∗*^*P* ≤ 0.05 (treatments versus model group). ZZAE: *Zanthoxylum zanthoxyloides* alkaloidal extract.

There was no significant difference in the levels of electrolytes (serum chloride, calcium, and sodium) and blood pH with respect to the control, model, and ZZAE groups. However, urea, creatinine, and potassium levels increased in the model group compared to the control group. Interestingly, ZZAE significantly (*P* < 0.05) decreased elevated urea and creatinine levels compared to the model group (Figure 6).

### 3.7. Effect of ZZAE on Blood Profile

**Figure 7:**
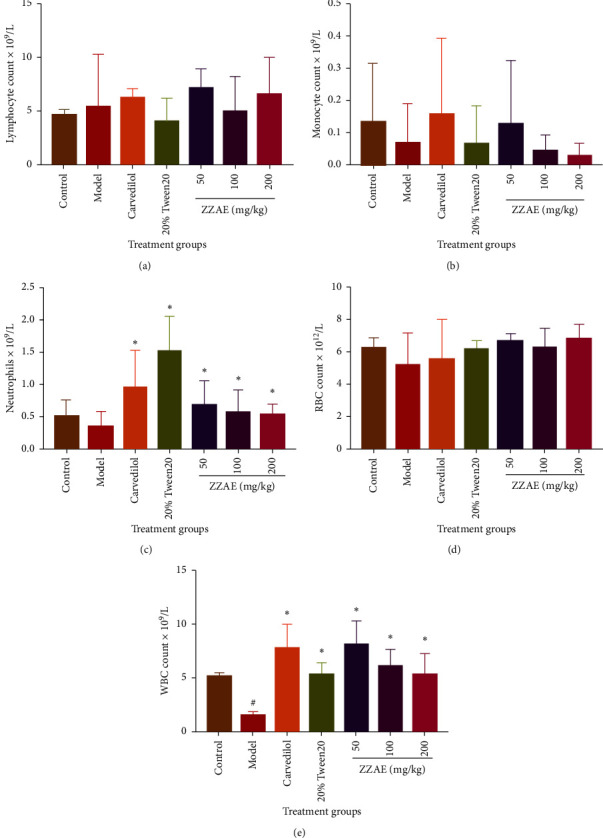
Effect of treatments on full blood count (FBC). (a) Lymphocyte count, (b) monocyte count, (c) neutrophils count, (d) red blood cell (RBC) count, and (e) white blood cell (WBC) count. Each value is the mean ± SD, *n* = 4. ^#^*P* ≤ 0.05 (control versus model group); ^*∗*^*P* ≤ 0.05 (treatments versus model group). ZZAE: *Zanthoxylum zanthoxyloides* alkaloidal extract.

Compared to the control group, the model group decreased monocyte, neutrophils, RBC, and WBC counts. However, ZZAE reversed these parameters (monocyte, neutrophils, RBC, and WBC) relative to the model group. The model group demonstrated increased lymphocyte count compared to the control group. However, ZZAE (200 mg/kg) decreased lymphocyte count relative to that of the model group (Figure 7).

### 3.8. Prophylactic and Oral Safety of ZZAE

**Figure 8:**
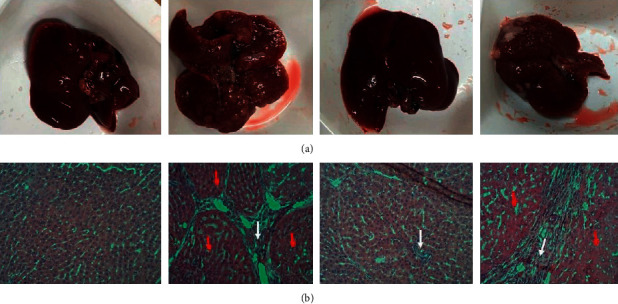
Prophylactic and oral safety of ZZAE. Control group (AI and BI), model group (AII and BII), ZZAE only group (AIII and BIII), and prophylactic ZZAE group (AIV and BIV). ZZAE: *Zanthoxylum zanthoxyloides* alkaloidal extract.

Compared to the model group (Figure 8, AII and BII), control (Figure 8, AI and BI) livers showed normal hepatic macroscopic and microarchitecture, while model livers showed accumulation of fatty tissues, inflammatory cell infiltration, intense necrosis (white arrow), and fibrogenesis (red arrows). ZZAE only group demonstrated hepatic macro- and microarchitecture similar to control livers (Figure 8, AII and BIII). Compared to model livers, prophylactic ZZAE (Figure 8, AIV and BIV) improved the degenerated hepatic macro- and microarchitecture. Compared to the model group, ZZAE only and prophylactic ZZAE improved tumor multiplicity and also survival of rats (Table 5).

### 3.9. ZZAE Slows Growth of *A. cepa* Roots

**Figure 9:**
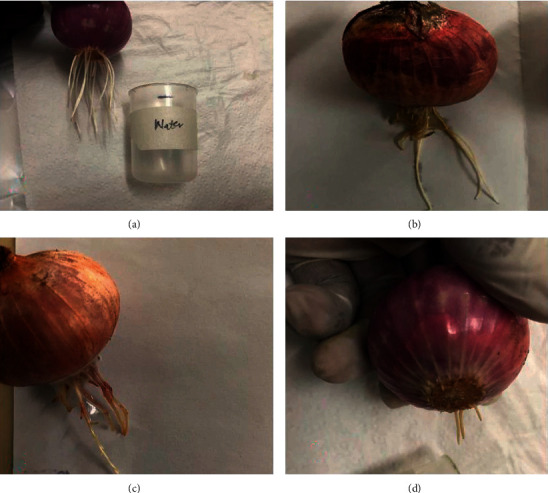
Effect of ZZAE on the growth of *A. cepa* roots. (a) *A. cepa* roots grown in water, (b) *A. cepa* roots grown in carvedilol (1.0 mg/mL), (c) *A. cepa* roots grown in ZZAE (0.1 mg/mL), and (d) *A. cepa* roots grown in ZZAE (1 mg/mL).

Compared to control (onion roots grown in distilled water), ZZAE concentration dependently inhibited the growth of onion roots (Figure 9). *A. cepa* roots grown in distilled water had a mean root length of 4.7 ± 0.8 cm compared to that of carvedilol (2.6 ± 1.4 cm), ZZAE (2.9 ± 0.4 cm), and ZZAE (0.6 ± 0.2 cm) over 72 hours.

## 4. Discussion

This study demonstrates the antihepatocellular carcinoma (HCC) and hepatoprotective effects of *Zanthoxylum zanthoxyloides* leaves in a chemically induced model of HCC in rats. Rejuvenated popularity and reliance on the use of medicinal plants as complementary and alternative medicine are premised on deficiencies (high recurrence, poor survival posttreatment, poor quality of life posttreatment, treatment-related complications, and cancer cell resistance) associated with conventional anticancer therapies. Previously, it was reported that plant species in the genus *Zanthoxylum* contain diverse bioactive anticancer compounds. In response, anticancer properties of the fruits of two (*Zanthoxylum zanthoxyloides* and *Zanthoxylum leprieurii*) species in the genus Zanthoxylum were assessed on leukemia and breast cancer cell lines [32, 33] and the results were promising. Subsequently, antiproliferative effects of the fruits of *Zanthoxylum zanthoxyloides* and *Zanthoxylum leprieurii* were investigated on a number of humanized cancer cell lines including liver (WRL-68), prostate (PC-3), breast (MCF-7), and colon (CaCO_2_) [34] and again the results were promising. Further, it was shown that terpenoids, alkaloids (aporphines, benzophenanthridines, and furoquinolines), acridone alkaloids, aromatic and aliphatic amides, coumarins, and lignans were present in fruit extracts of *Zanthoxylum zanthoxyloides* and *Zanthoxylum leprieurii* [34–36] and these phytocompounds could be responsible for some of the biological activities such as anti-inflammatory and gastroprotective effects of these two (*Zanthoxylum zanthoxyloides* and *Zanthoxylum leprieurii*) medicinal plants [12, 37].

The present study investigated anti-HCC and hepatoprotective effects of *Zanthoxylum zanthoxyloides* alkaloidal extract (ZZAE) *in vivo* and the results show that ZZAE at increasing doses decreased CCl_4_-induced liver injury from advancing from chronic liver fibrosis to HCC-like phenotype. Carbon tetrachloride (CCl_4_) is one of the commonly used hepatotoxins in chemical carcinogenesis, specifically to induce chronic liver injury in experimental rodents [38]. CCl_4_ is an indirect carcinogen; therefore, it must undergo enzyme-mediated biotransformation leading to formation of a potent hepatotoxic secondary metabolite (●CCl_3_). The chemically unstable metabolite abstracts oxygen leading to the formation of trichloromethyl peroxy (●OOCCl_3_) [39]. The ●OOCCl_3_ induces decrease in oxygen partial pressure that triggers oxidative stress, cellular damage, inflammatory reaction, and onset of liver fibrosis [40, 41]. ZZAE treatment might have disrupted the CCl_4_-mediated oxidative stress probably due to its phenolic and flavonoid components (Table 2) which are known natural antioxidants [42].

Pathologically, exposure of rats to CCl_4_ is characterized by fatty deposits around hepatic stellate cells, distortions of hepatic microstructure, infiltration of inflammatory cells, excessive deposition of collagen, loss of hepatocytes, and necrosis [43]. These observations were prominent in livers of rats exposed to CCl_4_ for 16 weeks as previously reported [44]. However, 12 weeks of treatment of CCl_4_-treated rats with ZZAE produced a dose-dependent recovery of the CCl_4_-mediated deteriorated liver microstructure and this observation mirrors an earlier report [45]. Also, CCl_4_-treated rats demonstrated a higher organ/body weight ratio, but a lower % change in body weight compared to ZZAE-treated rats and these observations was attributable to feed and water intake, which were low among CCl_4_-treated rats. The disconcordance observed with respect to organ/body weight ratio and %weight gain in CCl_4_-treated rats was probably due to hepatomegaly and hepatic edema which were common in CCl_4_-treated rats but hardly seen in ZZAE-treated rats. A 100% survival rate was observed among ZZAE-treated groups compared to CCl_4_-treated group and this observation was expected in view of the significant hepatoprotection demonstrated by ZZAE treatment (Figure 2; Table 3).

Biochemically, serum liver markers are routinely used to assess the functional status of the liver and also to assess responses of the liver to both acute and chronic injury [46]. Normally, elevated ALT and AST above control or bench ranges are considered to be associated with deteriorating liver injury [47]. Similarly, *γ*-glutamyltransferase (GGT) is used as an index of liver dysfunction and marker of alcohol intake [48]. There is a functional relationship between the liver and the kidneys in view of the fact that both organs are involved in excretion and in part metabolism of xenobiotics. Key markers of renal function aside from serum electrolytes include urea and creatinine. Elevated serum levels of urea and creatinine do not necessarily predict worsening kidney function, and neither should their levels within normal range be taken as an absence of renal dysfunction because the renal function is also influenced by extrarenal factors [49]. From this study, there were no significant differences between CCl_4_-treated rats and ZZAE-treated rats with respect to the studied renal markers (Figure 6) except serum urea and creatinine. Although CCl_4_-treated rats had elevated serum urea and creatinine, ZZAE treatment of rats exposed to CCl_4_ produced a significant decrease in the levels of serum urea and creatinine, perhaps suggesting improvement in the functional status of the liver and the kidney.

The bioactivity of pharmacological agents traditionally reflects their chemical composition and the diversity of functional groups present. From this study, phytochemical screening of *Zanthoxylum zanthoxyloides* extract (ZZE) revealed terpenoids, alkaloids, tannins, saponins, flavonoids, and phenols, an observation which agrees with earlier reports on extracts of *Zanthoxylum* spp. [34, 35, 50]. Further fractionation of ZZE to obtain the crude alkaloid (ZZAE) which was subjected to HPLC analysis indicated six revealing peaks (Figure 1) worth characterizing. The study could have benefited from isolating each of the six individual alkaloids; nonetheless, the present results do not only provide the needed rationale for future studies to separate the prominent peak fractions of ZZAE to obtain pure individual fractions but also confirm earlier reports on *Zanthoxylum zanthoxyloides* which indicate that it has many bioactive anticancer compounds [21, 34].

Put together the present study has demonstrated hepatoprotective effects of ZZAE in a rat model of HCC, and this finding in part substantiates not only the hypothesis “medicinal plants with demonstrated anti-inflammatory properties could demonstrate anticancer effects against inflammation-related cancers such as HCC” but also that Z. *zanthoxyloides* indeed contains bioactive anticancer compounds, mostly alkaloids including fagaroine [51].

## 5. Conclusion

ZZAE has demonstrated the anti-HCC and hepatoprotective effects to reverse HCC-like phenotypes in CCl_4_/olive oil-induced HCC rats and this finding rationalizes the need to further investigate ZZAE for specific anticancer compounds.

## Figures and Tables

**Table 1:** Yield of *Zanthoxylum zanthoxyloides* alkaloidal extract (ZZAE) and *Zanthoxylum zanthoxyloides* extract (ZZE).

Initial weight of dried leaves (g)	Yield of ZZE (g)	% yield of ZZE^a^	Yield of ZZAE (g)	% yield of ZZAE^b^
4,371.173	762.84	17.45	119.349	2.73

^a^(yield of ZZE (g)/initial weight of dry leaves (g) ^*∗*^100); ^b^(yield of ZZAE (g)/yield of ZZE (g) ^*∗*^100).

**Table 2:** Phytochemical composition of ZZE.

Phytocompound	Phytochemical test	Results
Terpenoids	Salkowski test	**+**
Carbohydrates	Molisch's test	**+**
^*∗*^Alkaloids	Dragendorff's test	**+**
Saponins	Foam test	**+**
Tannins	Lead acetate test	**+**
Flavonoids	Sodium hydroxide test	**+**
Phenols	Folin–Ciocalteu	−

^*∗*^Informed extraction of ZZAE; + = present; − = absent; ZZE: *Zanthoxylum zanthoxyloides* extract.

**Table 3:** Effect of ZZAE on body weight changes.

Treatment groups	Initial mean body weight (g)	Final mean body weight (g)	Change in mean body weight (g)	% change in mean body weight
Control	182.851 ± 10.39	274.448 ± 9.125	91.60 ± 9.002	50.1
Model	196.074 ± 7.245	224.990 ± 4.093	28.92 ± 8.357^#^	14.74
Carvedilol	174.803 ± 5.39	227.275 ± 3.224	52.472 ± 4.624^*∗*^	30.01
^a^Tween20	177.005 ± 3.689	209.336 ± 4.579	32.331 ± 6.353^*∗*^	18.26

ZZAE (mg/kg)				
50	182.446 ± 7.576	239.648 ± 6.397	57.202 ± 7.788^*∗*^	31.35
100	177.121 ± 3.58	242.089 ± 9.771	64.968 ± 9.753^*∗*^	36.68
200	184.874 ± 3.331	233.421 ± 4.243	48.547 ± 3.24^*∗*^	26.26

^#^ *P* ≤ 0.05 (control versus model groups); ^*∗*^*P* ≤ 0.05 (model versus all other treatment groups); ^a^(vehicle, 1 mL/rat, once daily).

**Table 4:** Effect of ZZAE on tumor incidence.

Treatment groups	^*α*^Tumor incidence
Control	0 to 5 ± 0.0
Model	˃15 ± 3.5^#^
^a^Tween20	˃15 ± 2.8^*∗*^
Carvedilol (6.25 mg/kg)	10 to 15 ± 2.8^*∗*^

ZZAE (mg/kg)	
50	10 to 15 ± 2.3^*∗*^
100	6 to 10 ± 0.8^*∗*^
200	6 to 10 ± 1.7^*∗*^

Each value is the mean ± SD, *n* = 4. ^#^*P* ≤ 0.05 (control versus model group); ^*∗*^*P* ≤ 0.05 (treatments versus model group); ZZAE: *Zanthoxylum zanthoxyloides* alkaloidal extract; ^*α*^determined by finding the average number of tumors counted by three independent researchers for each treatment group; ^a^vehicle for dissolution of ZZAE, 1 ml/rat.

**Table 5:** Effect of ZZAE preexposure on CCl_4_/olive oil-induced HCC.

Treatment groups	Change in mean body weight (g)	Change in mean liver weight (g)	Tumor multiplicity	Survival rate (%)
Control	91.60 ± 9.002	6.967 ± 0.412	0 to 5 ± 0.0	100
Model^a^	28.92 ± 8.357^#^	13.89 ± 1.759^#^	˃15 ± 3.5^#^	50
ZZAE only	83.065 ± 8.261^*∗*^	7.11 ± 0.481^*∗*^	0 to 5 ± 0.0^*∗*^	100
ZZAE^b^	73.299 ± 1.567^*∗*^	9.453 ± 0.717^*∗*^	0 to 5 ± 0.0^*∗*^	100

^a^Received CCl_4_-reconstituted in olive oil (1 : 1 v/v) (2 ml/kg ip twice a week for 16 weeks); ^b^received ZZAE (100 mg/kg *po*) before CCl_4_/olive oil (1 : 1 v/v; 2 ml/kg ip; twice a week for 16 weeks); ^#^*P* ≤ 0.05 (control versus model group); ^*∗*^*P* ≤ 0.05 (model versus other treatment groups) (ZZAE only and prophylactic ZZAE). ZZAE: *Zanthoxylum zanthoxyloides* alkaloidal extract.

## Data Availability

Data are available from the corresponding author upon request.
